# DNA methylation-based chromatin compartments and ChIP-seq profiles reveal transcriptional drivers of prostate carcinogenesis

**DOI:** 10.1186/s13073-017-0443-z

**Published:** 2017-06-07

**Authors:** Poppy Simmonds, Erick Loomis, Edward Curry

**Affiliations:** 1 Division of Cancer, Imperial College London, Hammersmith Hospital, Du Cane Road, London, W12 0NN UK; 2 Centre for Cell, Gene & Tissue Therapeutics, UCL Medical School, Royal Free Hospital, Pond Street, London, NW3 2QG UK; 3 Helix, 1 Circle Star Way, San Carlos, CA 94070 USA

**Keywords:** Prostate cancer, Epigenetics, Transcriptional regulation, Chromatin compartments

## Abstract

**Background:**

Profiles of DNA methylation of many tissues relevant in human disease have been obtained from microarrays and are publicly available. These can be used to generate maps of chromatin compartmentalization, demarcating open and closed chromatin across the genome. Additionally, large sets of genome-wide transcription factor binding profiles have been made available thanks to ChIP-seq technology.

**Methods:**

We have identified genomic regions with altered chromatin compartmentalization in prostate adenocarcinoma tissue relative to normal prostate tissue, using DNA methylation microarray data from The Cancer Genome Atlas. DNA binding profiles from the Encyclopedia of DNA Elements (ENCODE) ChIP-seq studies have been systematically screened to find transcription factors with inferred DNA binding sites located in discordantly open/closed chromatin in malignant tissue (compared with non-cancer control tissue). We have combined this with tests for corresponding up-/downregulation of the transcription factors’ putative target genes to obtain an integrated measure of cancer-specific regulatory activity to identify likely transcriptional drivers of prostate cancer.

**Results:**

Generally, we find that the degree to which transcription factors preferentially bind regions of chromatin that become more accessible during prostate carcinogenesis is significantly associated to the level of systematic upregulation of their targets, at the level of gene expression. Our approach has yielded 11 transcription factors that show strong cancer-specific transcriptional activation of targets, including the novel candidates KAT2A and TRIM28, alongside established drivers of prostate cancer MYC, ETS1, GABP and YY1.

**Conclusions:**

This approach to integrated epigenetic and transcriptional profiling using publicly available data represents a cheap and powerful technique for identifying potential drivers of human disease. In our application to prostate adenocarcinoma data, the fact that well-known drivers are amongst the top candidates suggests that the discovery of novel candidate drivers may unlock pathways to future medicines.

Data download instructions and code to reproduce this work are available at GitHub under ‘edcurry/PRAD-compartments’.

**Electronic supplementary material:**

The online version of this article (doi:10.1186/s13073-017-0443-z) contains supplementary material, which is available to authorized users.

## Background

Study into the mammalian nucleus has revealed that higher order chromatin structure involves organization of DNA into co-localized compartments, such that physical interactions between loci occur almost exclusively within the same compartment [1]. One compartment is associated with more open, accessible chromatin and a higher overall level of transcription, while more closed chromatin is found in the other compartment, at the nuclear periphery and around the nucleolus [2]. These spatial interactions are likely to bring enhancer and promoter sequences into close proximity, giving rise to transcription, and alternatively to bring silencers and repressor regions together in order to suppress transcription. The boundary between these compartments is thought to be genetically encoded, but this is not well understood [3].

Initial data on the partitioning of the genome into these two compartments have come from applications of the Hi-C technique, which uses chromosome conformation capture in conjunction with high-throughput DNA sequencing to identify pairs of loci that were physically proximal enough to be cross-linked with formaldehyde [4]. When a matrix of pairwise contact density is created, a clear pattern emerges in which all loci belong to one of two groups. For each group the likelihood of within-group interactions is high, but the likelihood of between-group interactions is low [1]. A recently published study has described a method for inferring chromatin compartmentalization from CpG methylation microarray data without the need for Hi-C data [5]. This method provides the opportunity to use publicly available DNA methylation datasets, more widely available than Hi-C data, to investigate the role of chromatin compartmentalization in human diseases, potentially highlighting previously unknown molecular characteristics that could pave the way to novel therapeutics.

Prostate cancer is the most common cancer in males in developed countries, resulting in more than 300,000 deaths worldwide in 2012 (Cancer Research UK (CRUK) statistics). While many localized cancers are cured with initial therapy or may be sufficiently indolent that no treatment is necessary, some will be aggressive with metastasis leading to death from the disease [6]. Recent attempts to characterize the molecular basis for prostate adenocarcinoma (PRAD) have identified recurrent genomic aberrations. These include fusions of androgen-regulated promoters (e.g. TMPRSS2) with members of the E26 transformation-specific (ETS) family of transcription factors (occurring in approximately 50% of tumours [7]) and point mutations of TP53, FOXA1, PTEN and SPOP [8]. Epigenetic aberrations in prostate cancer have been identified, including GSTP1 hypermethylation occurring in up to 70% of tumours [9], and few other candidates have yielded promising results for diagnostic or therapeutic tools [6, 10].

**Fig. 1:**
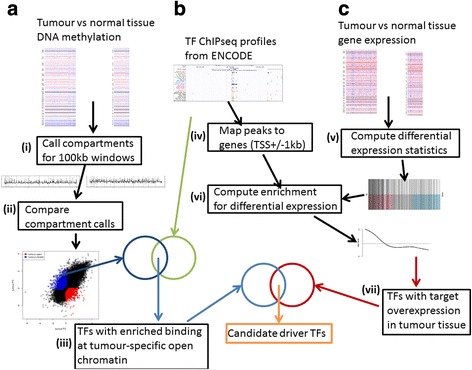
Schematic illustrating the overall analysis procedure, using three input datasets: **a** PRAD and normal prostate tissue DNA methylation data from The Cancer Genome Atlas (*TCGA*); **b** transcription factor (*TF*) DNA binding profiles from Encyclopedia of DNA Elements (*ENCODE*) ChIP-seq experiments; **c** PRAD and normal prostate tissue gene expression data from TCGA. Analytical steps are numbered (**i**–**vii**), with the final intersection between lists of TFs from steps (**iii**) and (**vii**) giving our candidate driver TFs

Importantly, despite considerable efforts, the molecular drivers remain unknown for approximately a quarter of all primary prostate cancers with both good and poor clinical prognosis [6]. Greater understanding of the molecular events driving prostate carcinogenesis and the mechanisms upon which the cancer cells depend for growth and metastasis could lead to the development of novel therapeutics that may succeed when standard treatments fail. To this end, we have applied chromatin compartmentalization methodology to DNA methylation profiles of primary PRADs and adjacent normal prostate tissue in order to find regions of aberrantly compartmentalized chromatin. We have integrated these profiles with gene expression profiles of the same tissue samples and with a compendium of experimentally derived genome-wide DNA binding profiles of transcription factors (TFs). Hypothesizing that cancer-driving TFs will have DNA binding sites in genomic regions which are aberrantly compartmentalized in addition to corresponding dysregulation of expression of downstream targets, this approach highlights both known and novel molecular drivers of PRADs. A graphical summary of our analytical approach is presented in Fig. 1.

## Methods

### Identifying regions of aberrant chromatin compartmentalization

Level 1 DNA methylation microarray data were downloaded from The Cancer Genome Atlas (TCGA) Data Portal. These data comprise two ‘idat’ files for each of 502 primary PRAD tissues and 50 normal prostate tissues. Idat files were processed in R using the ‘minfi’ package, according to the protocol in [5], using functional normalization and filtering out loci for which the probe sequence covers single nucleotide polymorphisms (SNPs) with minor allele frequency greater than 0.01. Regions with aberrant chromatin compartmentalization in PRAD were identified by comparing compartment calls obtained from analysis of the tumour tissue dataset against the compartment calls obtained from normal prostate dataset, for 100-kb windows tiled across the whole genome. Regions with a low confidence of compartment call as defined in [5] (numeric value reflecting confidence in compartment call less than 0.01) were excluded from subsequent analysis. All analyses described in this manuscript were restricted to chromosomes 1–22.

### Relating inferred compartment calls to chromatin accessibility and histone marks in a prostate cancer cell line

Chromatin accessibility data for the LNCaP prostate cancer cell line were downloaded from the Encyclopedia of DNA Elements (ENCODE), in the form of a normalized signal of unique reads from DNase-seq mapped to the hg19 human genome [ENCODE:ENCFF752YDY]. The sum of this signal of unique reads was computed for each 100-kb window, and these scores were compared for windows with different compartment calls as inferred from DNA methylation data.

Processed data as H3K27ac ChIP-seq peaks positions (called with Sole-Search [11], from [12]) mapped to the hg19 human genome were downloaded from Gene Expression Omnibus (GEO) [GEO:GSM1249448]. The proportion of each 100-kb window that is covered by an LNCaP H3K27ac peak was computed, and these scores were compared for windows with different compartment calls as inferred from DNA methylation data.

Processed data as H3K27me3 ChIP-seq peak summits (called with model-based analysis of ChIP-seq (MACS2) [13], from [14]) mapped to the hg19 human genome were downloaded from GEO [GEO:GSE86532]. Each 100-kb window was evaluated for overlap with an LNCaP H3K27me3 ChIP-seq summit, and the proportions of windows containing such a ChIP-seq summit were compared between the sets of windows with each compartment call inferred from DNA methylation data.

### Copy-number profiling of prostate adenocarcinoma tissues

Gene-level thresholded GISTIC2-processed copy-number data were downloaded from the University of California, Santa Cruz (UCSC) Cancer Browser website. Genes were mapped to each 100-kb genomic window using coordinates defined by Ensembl (Feb 2014 archive with hg19), and the total number of copy-number states (homozygous deletion, heterozygous loss, copy-neutral, low-level copy gain, high-level amplification) across the cohort was counted for each window.

### Mapping transcription factor binding sites

A compendium of TF binding sites was obtained from ENCODE representing 495 ChIP experiments with duplicates, covering 119 TFs in 77 human cell lines. Only peaks passing an irreproducible discovery rate (IDR) filter of 2% across at least two replicates were included as putative DNA binding sites. Androgen receptor binding sites were obtained from GEO [GEO:GSE65478], using data from [15]. Binding sites were provided as bed files denoting ChIP-seq peak coordinates in the hg19 human genome. TF ChIP-seq studies were then mapped to target genes for each gene with a ChIP-seq peak lying within 1 kb of its transcription start site (TSS) as defined by Ensembl (February 2014 archive with hg19).

### Assessing tumour-specific gene expression

Normalized gene-level counts for RNA-seq data for 500 primary PRAD tumours and 67 normal prostate tissues were downloaded from the UCSC Cancer Browser website. Empirical Bayes moderated *t* statistics for differential expression between tumour and normal samples were obtained using ‘limma’ [16].

### Identifying transcriptional drivers of PRAD

The fold enrichment of TFs binding DNA to regions of aberrant chromatin compartmentalization in PRAD was calculated as follows:

$$ F = \frac{\raisebox{1ex}{$ width\left( peaks{\displaystyle \cap } TSO\right)$}\!\left/ \!\raisebox{-1ex}{$ width\left( peaks/ TSO\right)$}\right.}{\raisebox{1ex}{$ width(TSO)$}\!\left/ \!\raisebox{-1ex}{$ width\left(\neg TSO\right)$}\right.} $$

where ‘peaks’ refers to the genomic regions showing reproducible TF binding in the ChIP-seq experiment; ‘TSO’ refers to the genomic regions with tumour-specific open chromatin; ‘$\neg T S O$’ refers to all genomic regions *without* tumour-specific open chromatin; ‘$peaks\cap T S O$’ denotes ChIP-seq peaks overlapping TSO regions; ‘$peaks/ T S O$’ denotes ChIP-seq peaks not overlapping TSO regions. A second analysis was repeated using a subset of the tumour-specific open chromatin regions comprising those regions which had both a change in sign of the principal eigenvector of the DNA methylation correlation matrix and also an absolute change in value of at least 0.2. This reflects greater confidence in the difference in compartment calls between tumour and normal tissues.

The statistical significance of differential expression of a TF target was assessed through rank sum tests implemented in the ‘geneSetTest’ function within the R package ‘limma’. This tests against the null hypothesis that the ranks of the TF target genes are randomly distributed across the differential expression statistics, from most overexpressed gene (in tumour relative to normal) to most underexpressed gene.

An integrated measure of tumour-specific chromatin accessibility was obtained by multiplying the logarithm (base 10) of the fold-change enrichment of the TF binding sites in tumour-specific open chromatin with –1 times the logarithm (base 10) of the *p* value for enrichment of the TF target genes in systematic overexpression in tumour samples relative to normal prostate tissues. TFs with a large positive integrated score were thus hypothesized to be likely transcriptional drivers of prostate adenocarcinoma, especially those TFs with relatively high-ranking scores in each individual characteristic.

A similar approach was used to find TFs with enrichment of binding sites in tumour-specific closed chromatin and systematic gene expression silencing in tumour samples relative to normal prostate tissues.

#### Assessing ***p***rostate-***s***pecific shRNA ***i***ncorporation/***d***epletion

Gene-level short hairpin (shRNA) selection biases in 216 cancer cell lines were downloaded from the Project Achilles portal (https://portals.broadinstitute.org/achilles). Prostate-specific lethality was tested by using a *t* test to compare the selection bias z-score in prostate cancer cell lines with the z-scores from all non-prostate cancer cell lines.

## Results

### Prostate adenocarcinomas have regions of aberrant chromatin compartmentalization across the genome

**Fig. 2:**
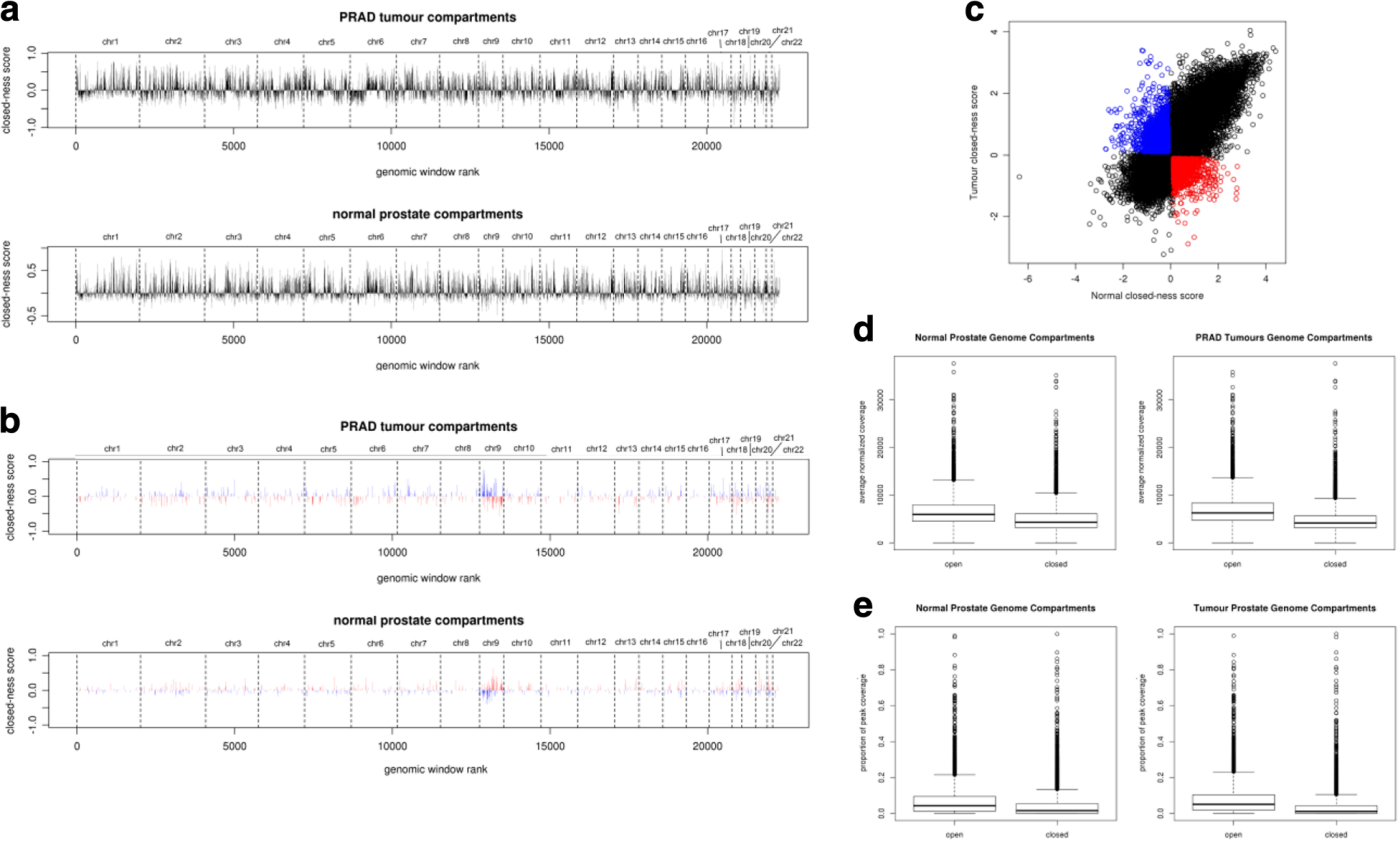
Genome-wide profiles of chromatin compartmentalization from PRAD and normal prostate tissues. **a** Genome-wide profiles showing assignment of 100-kb windows to open or closed chromatin compartments in PRADs (*top panel*) and in normal prostate tissue (*bottom panel*). *Horizontal axis* denotes rank of each 100-kb window by genomic coordinate, with each chromosome separated by *vertical dashed lines. Vertical axis* represents the confidence of assignment of each window to the closed compartment (+ve values) or the open compartment (–ve values). Confidence of assignment is based on the normalized value of the first eigenvector of the DNA methylation correlation matrix from each set of samples. **b** Scatter-plot showing the relationship between compartment assignment in PRAD tumours and in normal prostate tissue. *Horizontal axis* gives the closed-ness score (value in first eigenvector of the DNA methylation correlation matrix) for each 100-kb genomic window in the normal prostate tissues. *Vertical axis* gives the closed-ness score for the same genomic windows in the PRAD tumour tissues. After excluding windows with a low confidence assignment (defined as having a value of less than 0.1 in the first eigenvector of the DNA methylation correlation matrix), windows assigned to the open chromatin compartment in tumours but the closed chromatin compartment in normal tissue are plotted in *red*, while windows assigned to the closed chromatin compartment in tumours but the open chromatin compartment in normal tissue are plotted in *blue*. **c** Genome-wide profile of the differences in compartment assignment between PRAD tumours and normal prostate tissues, as in **a** but featuring only 100-kb windows with a different compartment assignment in the two tissue types. Genomic windows assigned to the open chromatin compartment in tumours but the closed chromatin compartment in normal tissue are plotted in *red*, while windows assigned to the closed chromatin compartment in tumours but the open chromatin compartment in normal tissue are plotted in *blue*. **d** DNA accessibility in LNCaP prostate cancer cell line, shown for genomic regions classified according to inferred compartment call from normal prostate and PRAD tumour DNA methylation data. Numerical values plotted show the distribution of the average DNAse I sequencing signal across each 100-kb window. **e** Accessible chromatin histone modification H3K27ac in LNCaP prostate cancer cell line, shown for genomic regions classified according to inferred compartment call from normal prostate and PRAD tumour DNA methylation data. Numerical values plotted show the distribution of the proportion of each 100-kb window that is covered by an H3K27ac ChIP-seq peak

DNA methylation microarray data were used to generate genome-wide chromatin compartmentalization maps for primary PRAD tumour and normal prostate tissue at a 100-kb resolution. The method described in [5] resulted in ‘open’/‘closed’ compartment calls for 100-kb windows tiled across the genome and a numeric value reflecting the degree of open-ness (the value of the first eigenvector of the correlation matrix). These compartment calls and scores were created for both tumour and normal tissue, and the corresponding profiles are shown in Fig. 2a. Compartment calls are available in Additional files 1 and 2. To confirm that these compartment calls inferred from DNA methylation data correspond to expected chromatin accessibility in prostate cancer cells, we analyzed a genome-wide profile of normalized DNase-seq signal from LNCaP cells (obtained from ENCODE). As the DNase-seq signal increases with chromatin accessibility [17], we compared the total signal in each 100-kb window of the chromatin compartmentalization maps to the corresponding compartment call. Genomic windows assigned to the open compartment had a significantly higher LNCaP DNase-seq signal than windows assigned to the closed compartment, both for the compartment calls inferred from prostate cancer tissues and those inferred from normal prostate tissue (*t* test *p* < 2 × 10^-16^, Fig. 2d). The difference between the median DNase-seq signal in the open and closed compartments inferred from prostate cancer tissues was 2126, whereas the equivalent difference for open and closed compartments inferred from normal prostate tissues was 1635. This implies that the compartment calls from the prostate cancer tissues correspond more closely to the chromatin accessibility of a prostate cancer cell line than the compartment calls from normal prostate tissues. We also compared regions of the genome with inferred open and closed chromatin compartments for their prevalence of peaks from ChIP-seq studies profiling active (H3K27ac) and repressive (H3K27me3) histone marks in the LNCaP cell line. Figure 2e shows the distribution of the proportions of 100-kb windows covered by H3K27ac peaks, separated into open and closed chromatin compartments inferred from the DNA methylation data. The median proportion of windows covered by an H3K27ac peak in the tumour open chromatin compartments was 0.05, but it was only 0.01 for windows in the tumour closed chromatin compartment. The equivalent values for windows separated by normal prostate chromatin compartments were 0.04 (open) and 0.02 (closed). For the repressive histone mark H3K27me3, we compared the proportion of genomic windows containing a peak ‘summit’ (highest point of ChIP enrichment) for open compartment regions and for closed compartment regions. Of the windows assigned to the open chromatin compartment in the PRAD tumour samples, 4.6% contained an H3K27me3 summit, but this increased to 7.8% for windows assigned to the closed chromatin compartment. Equivalent values based on normal prostate chromatin compartments were 4.7% (open) and 7.7% (closed). These results, particularly relating to H3K27ac, again imply that the chromatin compartments inferred from PRAD tumour samples reflect the profile of histone marks measured in a prostate cancer cell line more closely than the compartments inferred from normal prostate tissue samples.

To confirm that these regions of interest are not driven by changes in copy number, for each 100-kb window we computed the distribution of copy-number calls across this cohort of tumours. The proportions of genes with each copy-number state in the thresholded GISTIC2 data (homozygous deletion, heterozygous loss, copy-neutral, low-level copy gain, high-level amplification) are very similar for the tumour-specific open chromatin windows and the remaining windows (Additional file 3).

The genome-wide profiles of inferred compartment calls are largely similar between the cancer and normal tissue datasets, with 17,145 out of 22,313 (76.8%) 100-kb windows sharing the same compartment call in both cancerous and normal tissue. The profiles made by the numeric values reflecting compartment open-ness were very highly correlated, with Pearson correlation coefficient 0.753. Furthermore, when we exclude 203 windows with a low confidence of compartment assignment in either tissue type (as defined in [5]), the compartment agreement is 81.5% and the correlation coefficient 0.774. The relationship between these scores is illustrated in Fig. 2c, with the regions of aberrant PRAD compartmentalization (defined as those with a different compartment call in tumour vs normal tissue, after excluding those with a low confidence of either call) highlighted. The distribution of these aberrantly compartmentalized windows across the genome is shown in Fig. 2b. It is clear from this chart that there is a greater density of aberrant compartmentalization on Chr9 compared with the rest of the genome. In fact, chromosome 21 also has a greater than twofold enrichment of aberrant compartmentalization (a full table of ratios of observed/expected numbers of aberrantly compartmentalized windows for each chromosome is given in Additional file 4). This chromosomal enrichment of aberrantly compartmentalized windows is very similar for both tumour-specific open chromatin and tumour-specific closed chromatin and may arise from epigenetic changes linked to frequent aneuploidy events [18].

To get a sense of systematic functional effects driven by this aberrant compartmentalization, ENSEMBL genes overlapping with each (open and closed) set of aberrantly compartmentalized genomic windows were identified. Annotated pathway terms from Consensus Path DB (CPDB) [19] were tested for enrichment in these sets of overlapping genes. Surprisingly, no pathways were significantly enriched in regions of tumour-specific closed chromatin. However, a number of pathways were found to be significantly enriched in regions of tumour-specific open chromatin; these are shown in Additional file 5. They includes known cancer-associated pathways ‘phosphatidylinositol phosphate metabolism’ [20], ‘prolactin receptor signalling’ [21] and ‘platelet-derived growth factor receptor (PDGFR)-beta signalling’ [22]. We sought to gain further translatable insight into the systematic alteration of chromatin compartmentalization in PRAD.

### A subset of transcription factors preferentially bind DNA in regions of aberrant PRAD chromatin compartmentalization

**Fig. 3:**
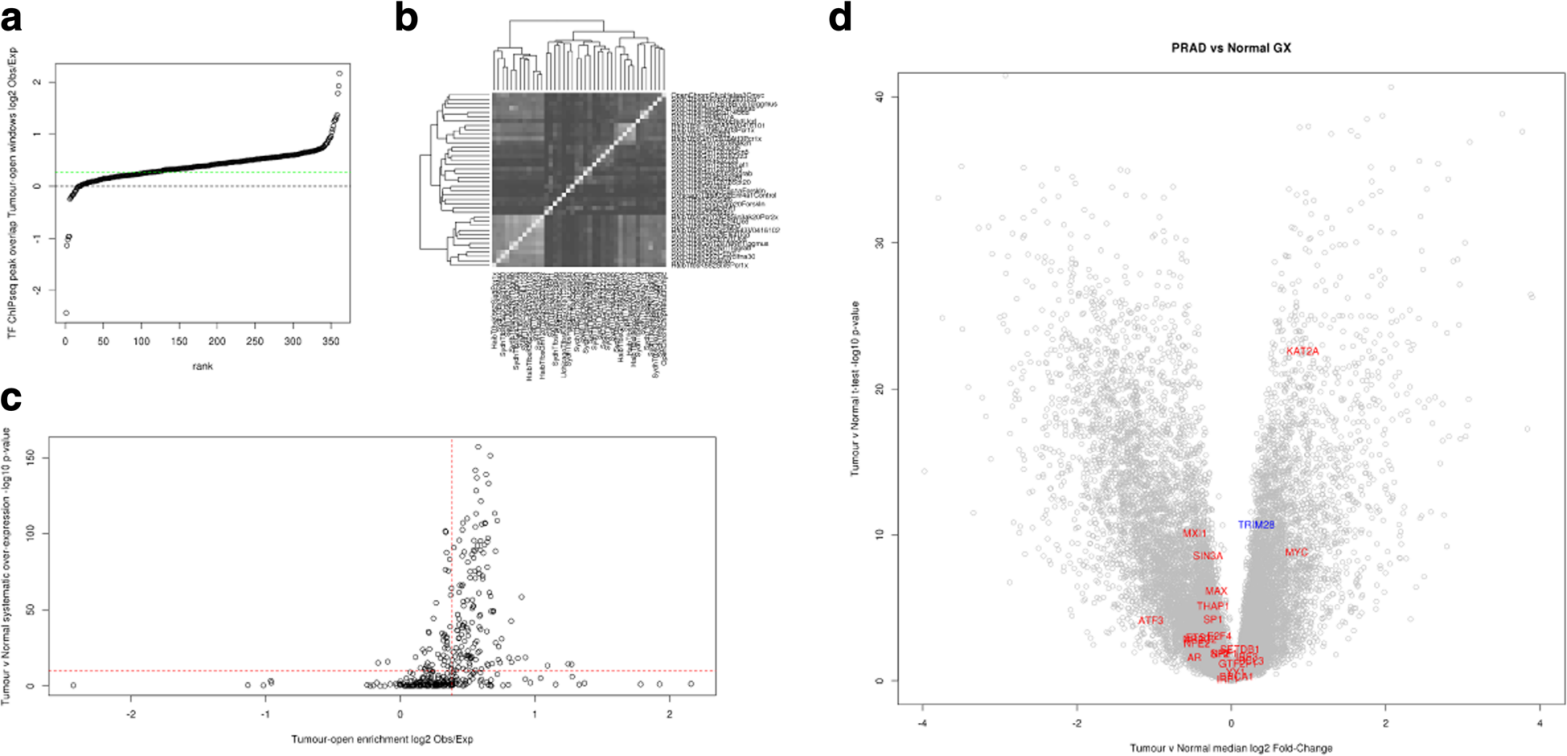
Analysis of transcription factor DNA binding profiles in the context of aberrant chromatin compartmentalization in prostate adenocarcinoma. **a** Degree of enrichment or depletion of TF ChIP-seq peak sites in the set of regions with PRAD-specific open chromatin compartmentalization. Each ENCODE ChIP-seq study has a corresponding data point, and they are ranked along the *horizontal axis* by the degree of enrichment (from most depleted on the *left* to most enriched on the *right*). *Vertical axis* gives log ratio of the observed over expected number of peaks lying in the regions of PRAD-specific open chromatin (i.e. windows assigned to the open chromatin compartment in tumours but the closed chromatin compartment in normal tissue). *Dotted horizontal line* at 0 indicates number of peaks in PRAD-specific open chromatin is exactly that expected by chance. **b** Heatmap showing overlap between peak locations for each of the most PRAD-specific open chromatin enriched TF ChIP-seq studies. *Black* indicates no overlap, *white* indicates total overlap. **c** Scatter-plot showing the relationship between a TF’s enrichment to PRAD-specific open chromatin and target gene overexpression in PRAD relative to normal prostate tissue. *Horizontal axis* gives log ratio of observed over expected number of peaks lying in PRAD-specific open chromatin (as in **a**) for each ENCODE TF ChIP-seq study. *Vertical axis* gives –log_10_ *p* value from test of systematic overexpression of inferred target genes of the corresponding TF (genes with ChIP-seq peak lying within 5 kb of TSS) in PRAD tumours relative to normal prostate tissues. *Dashed red lines* indicate the median value in each axis across all the included ENCODE TF ChIP-seq studies. **d** Volcano plot showing differential expression of candidate driver TFs in the context of all genes’ differential expression between PRAD tumours and normal prostate tissue. *Horizontal axis* gives log_2_ ratio between median expression in PRAD tumours and median expression across normal prostate tissues. *Vertical axis* gives –log_10_ *p* value of differential expression as evaluated through empirical Bayes moderated *t* test. Candidate drivers with ChIP-seq studies suggesting the TF’s DNA binding sites are enriched in regions of PRAD-specific open chromatin are shown in *red*, candidate driver (TRIM28) with ChIP-seq studies suggesting the TF’s DNA binding sites are enriched in regions of PRAD-specific closed chromatin is shown in *blue*

Hypothesizing that TFs critical to the malignant state of PRAD would have their DNA binding sites residing specifically in regions of aberrant chromatin compartmentalization, we used genome-wide TF binding profiles to identify likely drivers of the malignant characteristics of these tumours. This was achieved through evaluating the expected number of peaks from each ChIP-seq study lying in tumour-specific open chromatin windows and the expected number lying in tumour-specific closed chromatin windows. The ratio between observed and expected numbers was used to compute a fold enrichment for systematic co-localization of TF DNA binding sites to aberrantly compartmentalized chromatin in PRAD. The distribution of log fold changes is shown in Fig. 3a, which illustrates the fact that peaks from the majority of ENCODE TF ChIP-seq studies (regardless of the cell line in which the study was performed) are enriched for tumour-specific open chromatin compartments. Chi-squared tests to compare the observed distributions of peaks between tumour-specific open chromatin windows and all other windows revealed that most (409) of these enrichments were statistically significant after adjusting for multiple hypothesis tests. A table of log_2_ fold changes, chi-squared test *p* values and adjusted *p* values for all ChIP-seq studies, for both tumour-specific open chromatin and tumour-specific closed chromatin, is provided in Additional file 6.

To further evaluate the specificity of the TF binding site enrichment to tumour-specific open chromatin windows, we repeated this analysis with different selections of windows of interest. Varying the cutoff for filtering selected windows with low confidence of compartment assignment, and including the windows that were given the same compartment assignment in both tumour and normal tissues, we recomputed the fold enrichment for TF binding sites lying in the selected windows. A heatmap illustrating pairwise Spearman correlation coefficients between the profile of fold enrichment across the ChIP-seq studies for each selection of windows of interest is provided in Additional file 7. Enrichment scores of the TFs are similar across the range of cutoffs for sets of windows restricted to the same quadrant of Fig. 2c (e.g. both sets of windows are open in tumour tissue and closed in normal tissue, or both sets are open in tumour and open in normal tissue). Enrichment scores from one set of windows are generally dissimilar to those obtained using a set of windows that was in a different quadrant of Fig. 2c (e.g. one set of windows is open in tumour and closed in normal tissue, but the other set of windows is open in both tumour and normal tissue). This demonstrates the fact that the selection of TFs of interest with enrichment in tumour-specific open chromatin is not sensitive to the precise value of the cutoff used for filtering out windows with low confidence of compartment assignment. It also demonstrates that the enrichments specifically reflect the genomic regions with open chromatin in tumour and closed chromatin in normal tissue, and are not a result of a generic characteristic of DNA binding for those TFs. We also repeated the compartmentalization analysis using randomly sampled subsets (of equal size, *n* = 251) of tumour tissue samples for the two groups to compare. Firstly, the closed-ness scores inferred from each tumour subset were both markedly more correlated to the closed-ness scores inferred from the full tumour set than from the normal prostate tissue set (0.99 (tumour) vs 0.78 (normal) for the first subset, and 0.99 (tumour) vs 0.75 (normal) for the second). Second, to the two tumour subsets’ compartment scores we applied the approach previously used to identify 100-kb genomic regions with a clear difference in compartmentalization between two sets of compartment scores. This approach yielded 231 regions specifically open in one tumour subset vs the other, where the same threshold yielded 1582 regions specifically open in the tumours vs normal tissue. Of these, only 2 regions were shared across both analyses, which strongly implies that the genomic regions we have called tumour-specific open chromatin are not an artefact of our analytical approach and do indeed reflect the state of prostate tumour compartmentalization relative to normal prostate tissue.

To further test that the enrichments reflect properties of these specific sets of genomic regions, for each TF we evaluated the relationship between the log fold enrichment to tumour-specific open chromatin and the log fold enrichment to tumour-specific closed chromatin. The Spearman correlation coefficient across all TF studies was –0.524 (*p* < 2 × 10–^16^), which implies that an enrichment of a TF’s binding sites in tumour-specific open chromatin regions generally coincides with a depletion of that TF’s binding sites in tumour-specific closed chromatin.

As we were interested in establishing whether there may be different tumour-specific transcriptional modules being driven by groups of TFs, we computed the pairwise Jaccard distance to compare the lists of overlapping tumour-specific open chromatin windows for each ChIP-seq study. When these pairwise distances are plotted as a heatmap in Fig. 3b, the colours range from perfect overlap (white) to no overlap at all (black). We see that most of the TFs share a similar overall distribution of DNA binding sites, in that most of the off-diagonal blocks are light grey, but for nearly all TFs these are noticeably darker (indicating less overlap in binding site profiles) than the diagonal blocks (each TF compared to itself has perfect overlap). This implies that, while the different TFs may share common regions of DNA binding, they do have clear differences that could represent different parts of the tumour-specific transcriptional program. Knowing that some TFs have had ChIP-seq studies performed in multiple cell lines, we compared the tumour-specific open chromatin enrichment scores for ChIP-seq studies profiling the same TF with studies profiling different TFs. For a set of 14 TFs that were profiled in at least four different cell lines, we found that all had a smaller median same-TF difference than other-TF, regardless of the cell line (the table of values is provided in Additional file 8). This suggests that the enrichments we report are likely to reflect characteristics of the TFs in question, more than the specific cell lines in which the ENCODE ChIP-seq studies were performed.

### Integration of chromatin compartmentalization, gene expression and ChIP-seq data identifies known and novel transcriptional drivers of PRAD carcinogenesis

If a transcription factor were to drive a malignant phenotype through activation or repression of its downstream targets, one would expect these targets to have systematically altered levels of expression in tumours relative to normal tissue. In order to test this hypothesis, we used corresponding expression microarray data from the same cohort of TCGA samples as were profiled with DNA methylation microarrays and from which we inferred the chromatin compartmentalization calls. Using *t* statistics for differential expression between 500 PRAD tumours and 67 normal prostate tissue samples, we tested systematic enrichment towards overexpression in tumour for each TF’s target genes (defined as those with TSS within 1 kb of a ChIP-seq peak); these are presented in Additional file 9. When we compare each TF’s log_2_ enrichment towards tumour-specific chromatin and their –log_10_ *p* value representing degree of enrichment towards tumour overexpression of downstream target genes, we find a reassuring correlation (Pearson correlation coefficient = 0.45, *p* < 2.2 × 10^–16^). These values are shown in Fig. 3c, along with dashed lines indicating the median values of each statistic. In particular, we see that almost all of the TFs with greatest enrichment for overexpression in tumours also show clear enrichment of binding sites coinciding with regions of tumour-specific open chromatin. To ensure these results were robust to the choice of 1-kb cutoff for assignment of a gene to a given TF ChIP-seq study, we computed the corresponding –log_10_ *p* values using a range of cutoffs (1 kb, 2 kb, 5 kb, 10 kb). The resulting profiles of per-TF enrichment scores were compared by computing the Pearson correlation coefficient for each pair of cutoffs. That the smallest pairwise correlation coefficient was greater than 0.99 strongly suggests that the degree of PRAD-specific overexpression we have calculated is robust to the particular cutoff for assigning target genes to TFs.

We predict that these TFs have a key role to play in the pathogenesis of PRAD and as such merit further examination. To this end we looked at TFs for which ChIP-seq studies showed >1.5-fold enrichment to aberrantly open chromatin and systematic target gene overexpression with *p* > 1 × 10^–4^. Excluding RNAPolII (which would most likely just reflect actively transcribed genes in the corresponding ENCODE cell line), we find KAT2A, MYC, SIN3A, HEY1, SP2, MAX, YY1, ATF3, NRF1, BCL3, THAP1, MXI1, GABP and ETS1. These represent our top candidates for TFs driving aberrant gene expression in PRAD tumours. Looking back at the TCGA gene expression microarray data, we can see if any of the predicted driver TFs themselves show aberrant expression in the tumours, providing an obvious mechanism for the observed overexpression of their downstream targets. A volcano plot showing differential expression of these candidates in the context of all genes is presented in Fig. 3d. It is apparent that KAT2A and MYC are indeed overexpressed in PRAD tumours compared to normal prostate tissue. It is also clear that a number of the candidate drivers including MXI1, SIN3A, THAP1 and ATF3 are down-regulated in PRAD tumours. This suggests that these genes may represent transcriptional repressors that are themselves silenced in the malignant phenotype. Additionally, we find TRIM28 to be the only profiled TF significantly depleted for binding the tumour-specific open chromatin, significantly enriched in tumour-specific closed chromatin and with its target genes showing systematically down-regulated expression in PRAD tumours compared to normal prostate tissue. That TRIM28 itself is overexpressed suggests that it may be functioning as a transcriptional repressor actively promoting a malignancy-associated gene expression program. Although a number of the candidate TFs are not differentially expressed themselves between tumours and normal tissue, it does not rule out the possibility of them driving differences in gene expression programs, as their activity could be regulated through post-translational modifications or through differential expression of a co-factor required for DNA binding.

This set of putative drivers of aberrant transcription in PRAD tumours contains a number of known drivers of malignancy, either through hyperactivation or loss. Gain-of-function drivers include MYC [23], ETS1 [24], GABP [25], YY1 [26] and NRF1 [27]. Loss-of-function drivers include SIN3A [28], ATF3 [29], MXI1 [30] and THAP1 [31]. Amongst these sets, the novel candidates KAT2A, TRIM28 and HEY1 are particularly interesting, as they represent previously unknown putative drivers of prostate cancer. Further supporting this hypothesis in the case of KAT2A is the observation that KAT2A-targeting shRNAs were specifically depleted (*t* test *p* = 0.06) in terms of incorporation into prostate cancer cell lines relative to all other cancer cell lines in the Achilles high-throughput screen [32].

## Discussion

The approach taken in this study utilizes integration of a publicly available dataset of matched DNA methylation and gene expression profiles, together with a compendium of DNA-binding profiles, to identify transcriptional drivers of disease. To our knowledge, this is the first time that inference of spatial organization of chromatin has been used in such a way. This study therefore provides an example from which applications to other clinically relevant phenotypes could be undertaken, highlighting potentially therapeutically targetable pathways. To this end, we acknowledge that direct pharmacological targeting of TFs has historically been challenging [33]. We therefore propose using candidate driver TFs discovered through our approach as a focal point from which to search for regulating or interacting partners that may have more favourable characteristics for druggability.

The genomic regions of interest with tumour-specific open chromatin were identified from primary tissue samples; therefore, differences in the infiltration of different cell types across each of the two primary tissue cohorts could leave a signature in the correlations in the levels of CpG methylation, on which the compartment calls are based. As the potentially confounding cell types and their methylation profiles at the CpG sites included in the analysis (which excludes those in promoter CpG islands) are unknown, it is impossible at this stage to separate correlations due to varying levels of infiltrating cell types from the correlations that reflect the chromatin compartments. Given that our analysis of DNase-seq data shows that the compartment calls we have used for the work presented here do indeed reflect chromatin accessibility and associated histone modifications in a prostate cancer cell line, we believe that any effect of cell type composition in the primary tissues is not sufficient to detract from the assumed biological significance of the compartment calls.

We have assumed that TFs with clear evidence for preferential binding of DNA at regions of the genome undergoing spatial reorganization in a disease state are likely to be important for driving, or at least maintaining, that state. While it is out of the scope of this study to carry out experimental work needed to obtain direct evidence of the role of the candidate TFs highlighted through our application in prostate cancer, it is encouraging to have found considerable evidence in the literature. It is noticeable that a number of the candidates identified in our application to PRAD are linked to the oncogene MYC: notably MXI1, MAX and SIN3A. MXI1 forms a heterodimer with MAX which sequesters MAX away from MYC [34]. SIN3A directly deacetylates c-Myc protein, suppressing its activity [35]. That so many of the genes encoding well-known Myc-interacting proteins are found in this analysis could be due to the fact that MYC is such an active and important driver of prostate cancer that the whole genome is reorganized to make its downstream targets more accessible. As the genome-wide DNA binding profiles were obtained from ChIP-seq experiments in immortalized cell lines [36], they are likely to have downstream targets of MYC accessible for transcription and for pull-down during the ChIP procedure. ChIP with antibodies to any proteins binding MYC may be particularly likely to enrich similar regions of the genome, and hence likely to appear in any similar application of our analysis to compare rapidly proliferating cells with normal physiological tissue. Of the highlighted genes without previously known roles in prostate cancer, we find it interesting to note that both KAT2A and TRIM28 have epigenetic remodelling functions. KAT2A encodes an acetyltransferase which can alter the epigenetic state of promoters through acetylating histones [37]. TRIM28 (which encodes the KAP1 protein) is a master regulator of transcription implicated in the control of a wide range of biological processes [38]. Given that we propose KAT2A and MYC as candidate driver TFs in PRAD which are overexpressed in tumours relative to normal prostate tissue, one might expect to see a more ‘normal-like’ compartment profile in tumours with low KAT2A or MYC expression relative to those with high KAT2A and MYC expression. However, when we attempted this analysis using MYC-low or KAT2A-low subsets, the compartment calls were more dissimilar to both normal and tumour compartment calls than they were to each other. As MYC is such a ubiquitous oncogenic driver, we presume that heterogeneity within the MYC-low tumours or alternative mechanisms for activation of the MYC-driven transcriptional program are more likely reasons for this difference, rather than MYC not being a DNA-binding factor driving malignancy.

It would also require further experimental work to determine whether the identified TFs were behaving as ‘pioneer factors’ [39] and actively reorganizing the chromatin structure in order to enable gene expression, or if some other mechanism was reorganizing the chromatin and the TFs merely reflect focal points in the transcriptional programs that are being aberrantly activated in the disease state. This is based on a model in which TFs with the most enriched overlap of binding sites to regions of tumour-specific open chromatin are most likely to be able to bind accessible chromatin in prostate tumour cells and to keep that chromatin accessible. If a TF can bind specific genomic regions that lead to stable overexpression of a set of genes that give cells a selective advantage in the context of prostate cancer development, then this should be reflected in the enrichments regardless of the cell line or conditions in which the ChIP-seq study to profile the TF possible binding sites was performed. Our analysis in effect uses TFs (via their experimentally derived DNA binding profiles) to annotate the functional genomic landscape of the disease phenotype in relation to the normal physiological state. In that sense, the mechanism of chromatin reorganization is of lesser importance where the TFs are not acting as pioneer factors, as they are nonetheless required for the disease state and therefore point to novel therapeutic strategies for treating the disease in question.

## Conclusions

In this study we have shown how DNA methylation and gene expression datasets can be used in conjunction with ENCODE’s collection of DNA binding profiles from human TFs to find likely transcriptional drivers of a disease state. We have highlighted known and novel drivers of prostate adenocarcinoma, suggesting further investigation into the role of KAT2A and TRIM28 in carcinogenesis. Through this application we have demonstrated that, given the ready availability of such datasets, our approach represents a powerful technique for understanding the complex transcriptional dysregulation underlying carcinogenesis and identifying pathways of inquiry for novel therapeutic targets in cancer.

## Additional files

Additional file 1: Table of open/closed compartment calls for PRAD tumours in 100-kb genomic windows. (TXT 1267 kb)

Additional file 2: Table of open/closed compartment calls for normal prostate tissue in 100-kb genomic windows. (TXT 1267 kb)

Additional file 3: Bar chart showing distribution of copy-number calls across PRAD tumour cohort for tumour-specific open chromatin genomic windows and for all other windows. (PNG 45 kb)

Additional file 4: Ratios of observed/expected numbers of aberrantly compartmentalized windows for each chromosome. (TXT 532 bytes)

Additional file 5: List of pathways found to be significantly enriched in regions of tumour-specific open chromatin. (TXT 468 kb)

Additional file 6: Table of log_2_ fold changes, chi-squared test *p* values and adjusted *p* values for all ChIP-seq studies, for both tumour-specific open chromatin and tumour-specific closed chromatin. (TXT 50 kb)

Additional file 7: Heatmap illustrating correlation coefficients for per-TF profiles of enrichment to tumour-specific open chromatin, across a range of thresholds for excluding windows with low confidence of compartment assignment. (PNG 163 kb)

Additional file 8: Table of median differences in tumour-specific open chromatin enrichment log fold changes for sets of ChIP-seq studies profiling the same TFs in different cell lines, and different TFs in the same cell lines. (XLS 24 kb)

Additional file 9: Table of geneSetTest *p* values and adjusted *p* values for mapped TF targets from each ChIP-seq study, representing systematic over- or underexpression in PRAD tumour samples relative to normal prostate tissue. (TXT 69 kb)

## Abbreviations

ChIP-seq: Chromatin immunoprecipitation followed by high-throughput DNA sequencing
CPDB: Consensus path database
ENCODE: Encyclopedia of (regulatory) DNA Elements
IDR: Irreproducible discovery rate
PRAD: Prostate adenocarcinoma
TCGA: The Cancer Genome Atlas
TF: Transcription factor
TSS: Transcription start site
