# Supplementary figures and images for: DNA methylation-based chromatin compartments and ChIP-seq profiles reveal transcriptional drivers of prostate carcinogenesis
Source: Genome Med. 2017 Jun 7;9:54. doi: 10.1186/s13073-017-0443-z (PMC5463361; doi:10.1186/s13073-017-0443-z)

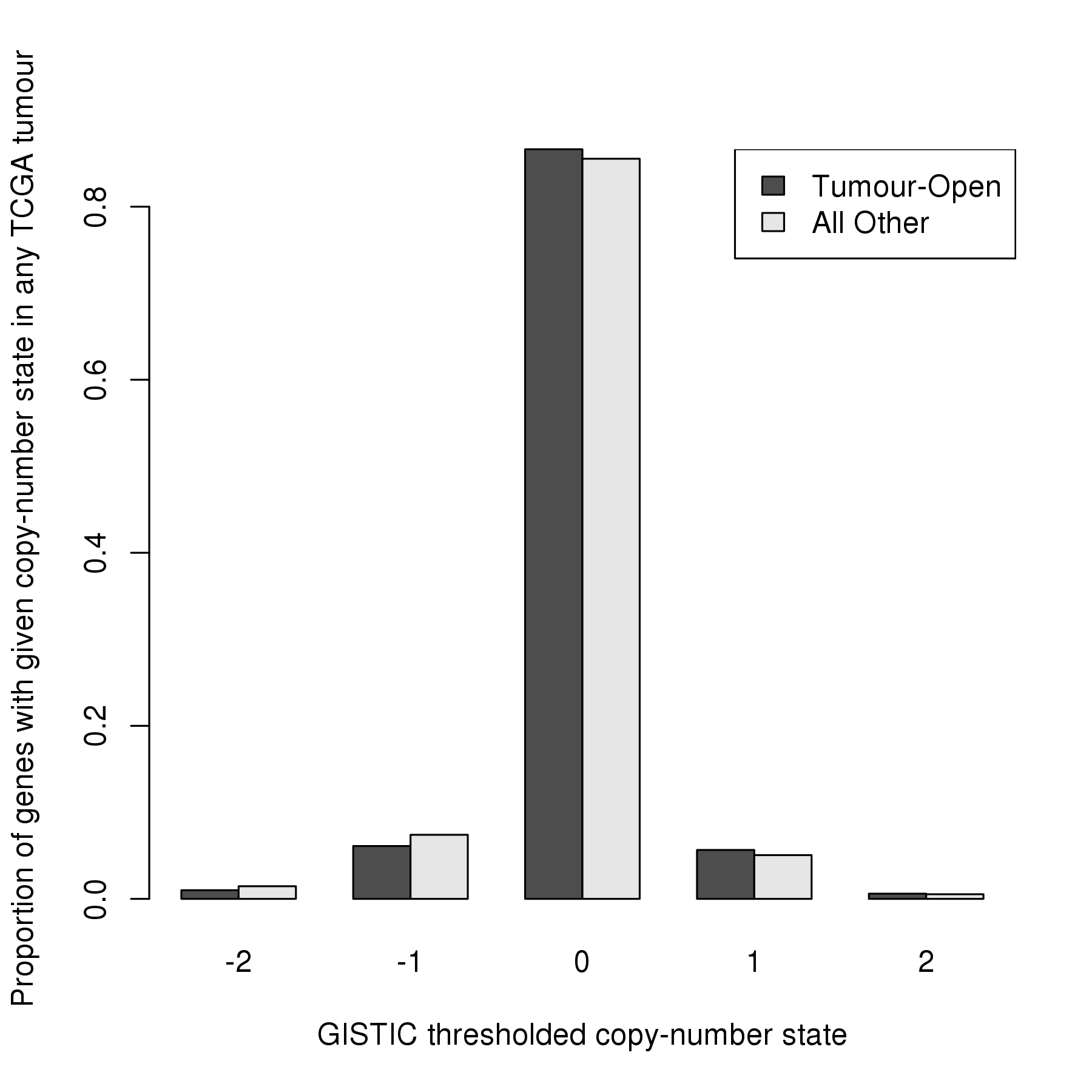

Supplement: Supplementary file 3 — Bar chart showing distribution of copy-number calls across PRAD tumour cohort for tumour-specific open chromatin genomic windows and for all other windows. (PNG 45 kb) [file 13073_2017_443_MOESM3_ESM.png]

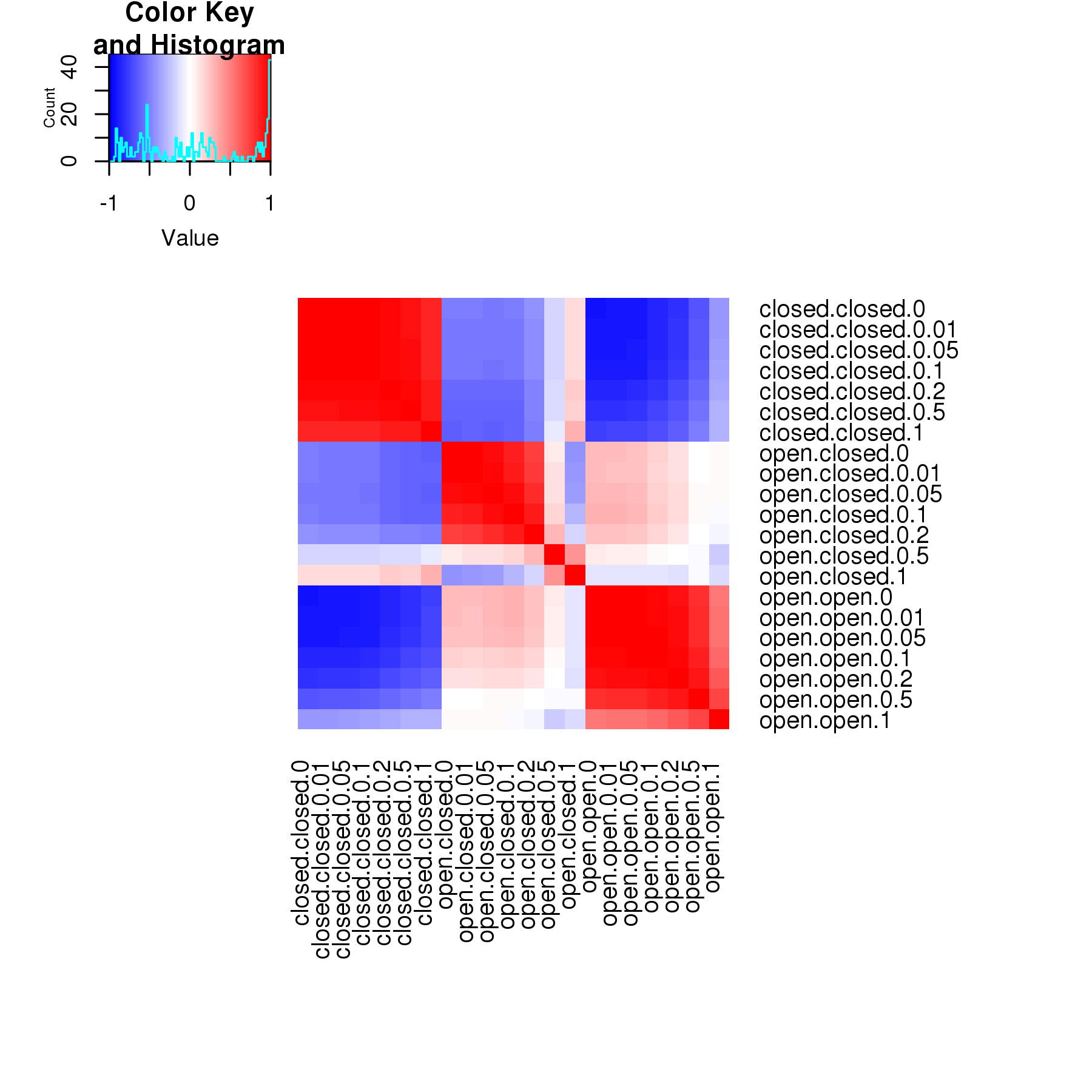

Supplement: Supplementary file 7 — Heatmap illustrating correlation coefficients for per-TF profiles of enrichment to tumour-specific open chromatin, across a range of thresholds for excluding windows with low confidence of compartment assignment. (PNG 163 kb) [file 13073_2017_443_MOESM7_ESM.png]
